# Advances in optical molecular imaging for neural visualization

**DOI:** 10.3389/fbioe.2023.1250594

**Published:** 2023-08-21

**Authors:** Jinzheng Wei, Chao Liu, Wenkai Liang, Xiaofeng Yang, Shufeng Han

**Affiliations:** ^1^ Department of Orthopaedics, First Hospital of Shanxi Medical University, Taiyuan, China; ^2^ First Clinical Medical College, Shanxi Medical University, Taiyuan, China; ^3^ Department of Urology, First Hospital of Shanxi Medical University, Taiyuan, China

**Keywords:** optical molecular imaging, multimodal imaging, intraoperative imaging, nerve visualization, neural fluorescence agents

## Abstract

Iatrogenic nerve injury is a significant complication in surgery, which can negatively impact patients’ quality of life. Currently, the main clinical neuroimaging methods, such as computed tomography, magnetic resonance imaging, and high-resolution ultrasonography, do not offer precise real-time positioning images for doctors during surgery. The clinical application of optical molecular imaging technology has led to the emergence of new concepts such as optical molecular imaging surgery, targeted surgery, and molecular-guided surgery. These advancements have made it possible to directly visualize surgical target areas, thereby providing a novel method for real-time identification of nerves during surgery planning. Unlike traditional white light imaging, optical molecular imaging technology enables precise positioning and identifies the cation of intraoperative nerves through the presentation of color images. Although a large number of experiments and data support its development, there are few reports on its actual clinical application. This paper summarizes the research results of optical molecular imaging technology and its ability to realize neural visualization. Additionally, it discusses the challenges neural visualization recognition faces and future development opportunities.

## 1 Introduction

The nervous system includes the central nervous system (CNS) and the peripheral nervous system (PNS), which take information from the surrounding environment and dominate the organism’s response to stimulus signals or stress. It controls muscle activity and glandular secretion and coordinates tissues and organs by transmitting signals and receiving feedback between body parts (Arafa et al., 2007; Carlstedt, 2008). The structural composition of the nerve varies from person to person, and the nerve is usually covered with a protective layer of tissue. Surgeons rely primarily on their anatomical knowledge, surgical expertise, and extensive clinical experience to identify important nerves during surgery. CNS or PNS damage produced by surgery may have devastating effects on a patient’s sensory, motor, and other bodily functions, negatively affecting the patient’s quality of life (Chaudhary et al., 2007; Shi et al., 2013; Musat et al., 2016). Hence, surgical planning frequently relies on imaging techniques such as computed tomography (CT), magnetic resonance imaging (MRI), and high-resolution ultrasonography (United States) to minimize the risk of nervous system injury during the procedure (Senden et al., 2006; Walsh et al., 2019). Intraoperative CT and MRI have the potential to provide valuable image guidance for surgeons. However, the operation of these systems can disrupt the normal surgical process, leading to prolonged surgery or anesthesia times. In addition, their expensive price and large floor space requirements can also impact their intraoperative use (Carl et al., 2018; Fuchs-Buder et al., 2018). In reagent applications, MRI can only identify the brain and spinal cord, but not peripheral nerve tissue due to the similar signal intensity between the PNS and surrounding tissues (Maravilla and Bowen, 1998). United States is a viable method for examining PNS, however, it is limited to superficial nerves due to its reliance on a network comprised of hypoechoic bands and hyperechoic lines (Misgeld and Kerschensteiner, 2006; Carvalho et al., 2019). The two most commonly utilized methods for testing nerve function are physical examination and electromyography (EMG). EMG is the prevailing approach for monitoring peripheral nerve function, achieved by stimulating target nerves to create action potentials (Kim et al., 2013; Barth and Gibbs, 2017). Based on current understanding, recognizing motor nerves is possible, but nonmotor sensory fibers are not recognized by the system (Walz et al., 2010). Although various techniques are available for nerve tissue treatment planning, they lack sensitivity and specificity, and some of them may pose radiological risks. Moreover, these techniques fail to achieve precise positioning of nerve tissue and anatomical details required for effective treatment planning.

In the report presented at the World Molecular Imaging Conference in 2009, Prof. Roger Yonchien Tsien first proposed the concept of optical molecular imaging-guided surgery. In 2011, the Leiden University Medical Center in the Netherlands and the Helmholtz Center Munich in Germany conducted a phase I clinical trial to evaluate the efficacy of their self-developed fluorescence imaging guidance system for the integrated treatment of peritoneal metastases in ovarian cancer (van Dam et al., 2011). In this field, the United States Food and Drug Administration (FDA) and the American Association for Image-Guided Surgery collaborated with 26 universities to establish standards. Additionally, the Canadian company Novadaq developed the first commercial real-time intraoperative imaging system called “Spy Fluorescence Imager,” which utilizes a CCD camera with filtering performance to achieve dynamic image acquisition (Liu et al., 2011). Subsequently, Roger Yonchien Tsien et al. successfully excised mouse tumor tissue using this technology proposed by their groups (Nguyen and Tsien, 2013). Xiaorong Xu’s team at the University of Science and Technology of China developed a miniaturized optical molecular image navigation eyepiece system by observing intraoperative fluorescence image information through a helmet display (Xu et al., 2014). In 2020, Jie Tian’s team at the Chinese Academy of Sciences, together with Sanjiv Sam Gambhir and Zhen Cheng’s team at Stanford University developed a near-infrared (NIR) I/II region imaging navigation system. This system achieved real-time multi-mode image detection on the micron level for the first time during the surgical resection of primary and metastatic liver tumors (Hu et al., 2020). In 2022, our team developed a solution for multi-mode three-dimensional optical molecular imaging intraoperative navigation imaging technology. This solution achieved a precise fusion of targeted optical molecular imaging and visible light images, accurately depicting the relationship between the three-dimensional structure of the target lesion and the surrounding tissue’s three-dimensional anatomy (Yang et al., 2022; Hao et al., 2023).

The advancement of optical molecular imaging technology has led to the emergence of new concepts such as optical molecular imaging surgery (Nguyen and Tsien, 2013; Wang et al., 2018), targeted surgery (Gandaglia et al., 2020), and molecular-guided surgery (Zhong et al., 2021; Mieog et al., 2022). These concepts involve the utilization of fluorescent dyes to label the target area, enabling real-time color contrast during surgical procedures. This technology utilizes fluorescent agents to mark target tissues and obtain real-time intraoperative images by irradiating them with light sources of varying wavelengths. It is capable of achieving high sensitivity at the skin level and high spatial resolution at the micron level, with the ability to capture hundreds of images per second. Fluorescence images provide a more intuitive representation of the tissue being imaged (Chi et al., 2014). The use of fluorescent agents to label nerve tissue has shown great potential in various surgeries. This technique enables surgeons to visualize nerves by observing optical signals hidden under the muscle or fat tissue during surgery, especially in situations where the normal anatomical position is displaced or destroyed due to external violence, which significantly reduces the difficulty of anatomy, maximizes the accuracy of the operation, shortens the duration of the operation, and minimizes the damage caused to the patient during the operation (Ietto et al., 2016).

**FIGURE 1:**
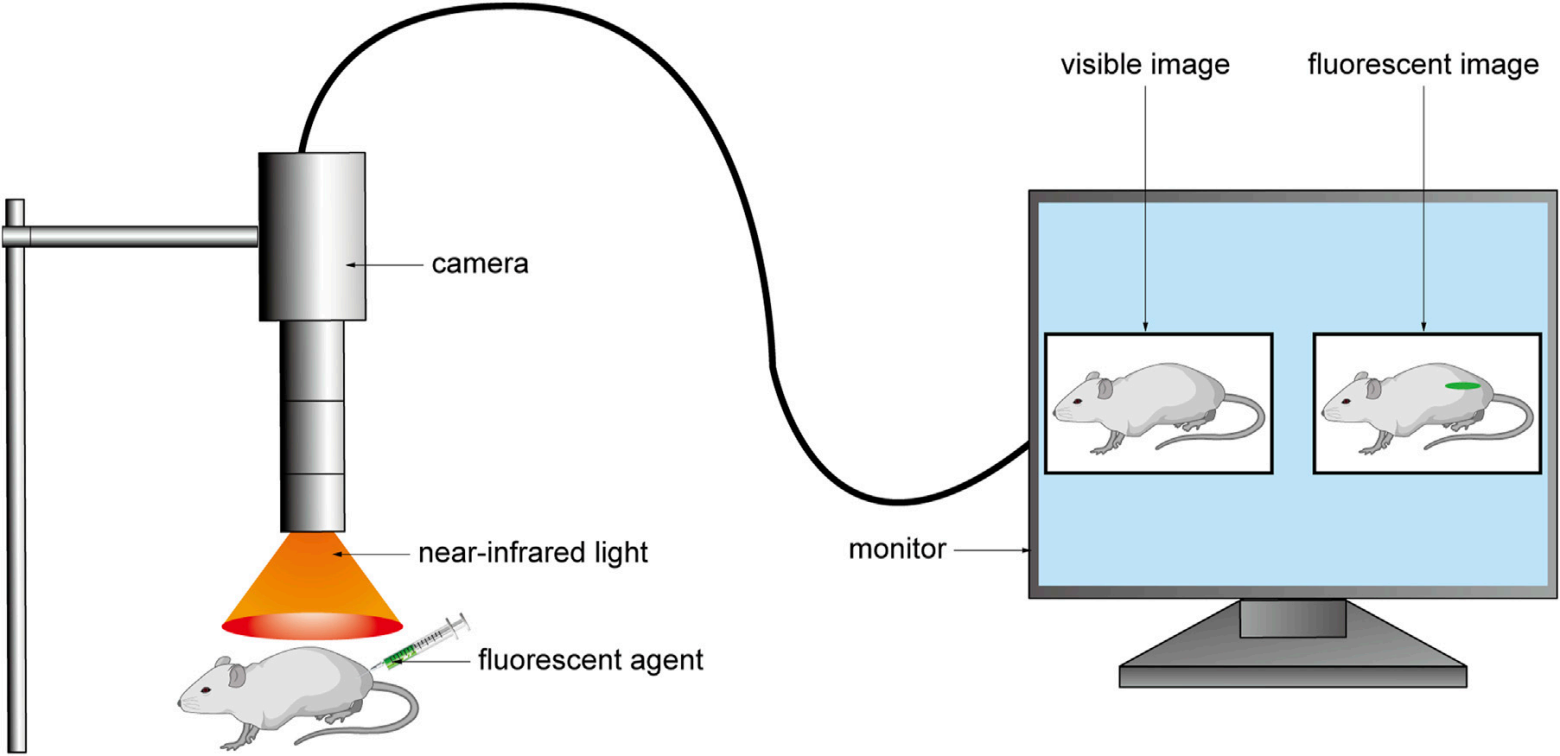
The fluorescent agent was injected into the body before the operation, and the fluorescent agent excited the fluorescent signal under the NIR light during the operation. The camera collected the signal and converted it into an image and transmitted it to the monitor to realize real-time intraoperative imaging.

Near-infrared light, an electromagnetic wave with a wavelength ranging from 700 nm to 2500 nm, is extensively employed in biological analysis research and biomedical diagnosis (Figure 1 (Beć et al., 2020)). Traditional optical imaging technology is mainly carried out in the spectral region of visible light (400–700 nm) and NIR I (700–900 nm) (Dip et al., 2014; Bennett et al., 2016; Lee et al., 2017; van Willigen et al., 2017; He et al., 2018; Du et al., 2021). However, when imaged in the visible region, the spatial resolution, imaging sensitivity, and contrast of these suffer from severe tissue scattering, absorption, and autofluorescence, leading to a decrease (Ren et al., 2021). NIR-I wavelengths are ideal for optical imaging as they can penetrate biological tissues such as skin and blood more deeply and effectively because they are not easily absorbed and scattered by these tissues (Prajapati et al., 2010; Vahrmeijer et al., 2013; Burggraaf et al., 2015). The autofluorescence of biological tissues in this wavelength range is minimal, resulting in a relatively high signal-to-background ratio (SBR) (Sakudo, 2016; Donnelly and Fine-Goulden, 2020). Currently, NIR-I wavelengths are the most suitable for deep tissue imaging of animals. Compared to NIR-I region optical imaging, the utilization of NIR-II (1,000–1700 nm) can result in lower levels of tissue autofluorescence, photon scattering, and absorption (Li et al., 2018; Li and Wang, 2018; He et al., 2019; Zhu et al., 2019). Additionally, it offers a higher SBR and can penetrate deeper into biological tissue (Li et al., 2020; Li et al., 2022b; Li et al., 2022a; Zhao et al., 2020; Zhao et al., 2021). Therefore, it is also regarded as the next-generation of optical imaging technology for assisting in biomedical diagnosis.

Since the beginning of the 21st century, optical molecular imaging has demonstrated both safety and effectiveness in numerous applications for surgical guidance (Tipirneni et al., 2017; Li et al., 2022). Medical devices utilizing this technology, particularly in the NIR region, are increasingly being approved for clinical use and hold a significant market share. These devices are currently capable of serving in endoscopic, laparoscopic, and open surgery procedures (Pogue and Rosenthal, 2021). With the advancement of imaging technology, the optical molecular imaging system has evolved to include independent imaging in visible light (Alfano et al., 1998) and NIR regions (Ntziachristos et al., 2003), bimodal imaging (Jathoul et al., 2014), multimodal imaging (Wang et al., 2020; Liu et al., 2022), 3D fluorescence imaging (Choi et al., 2020), color fusion imaging (Hao et al., 2023), and other modes. These improvements have significantly enhanced the doctor’s ability to visualize during operations.

**FIGURE 2:**
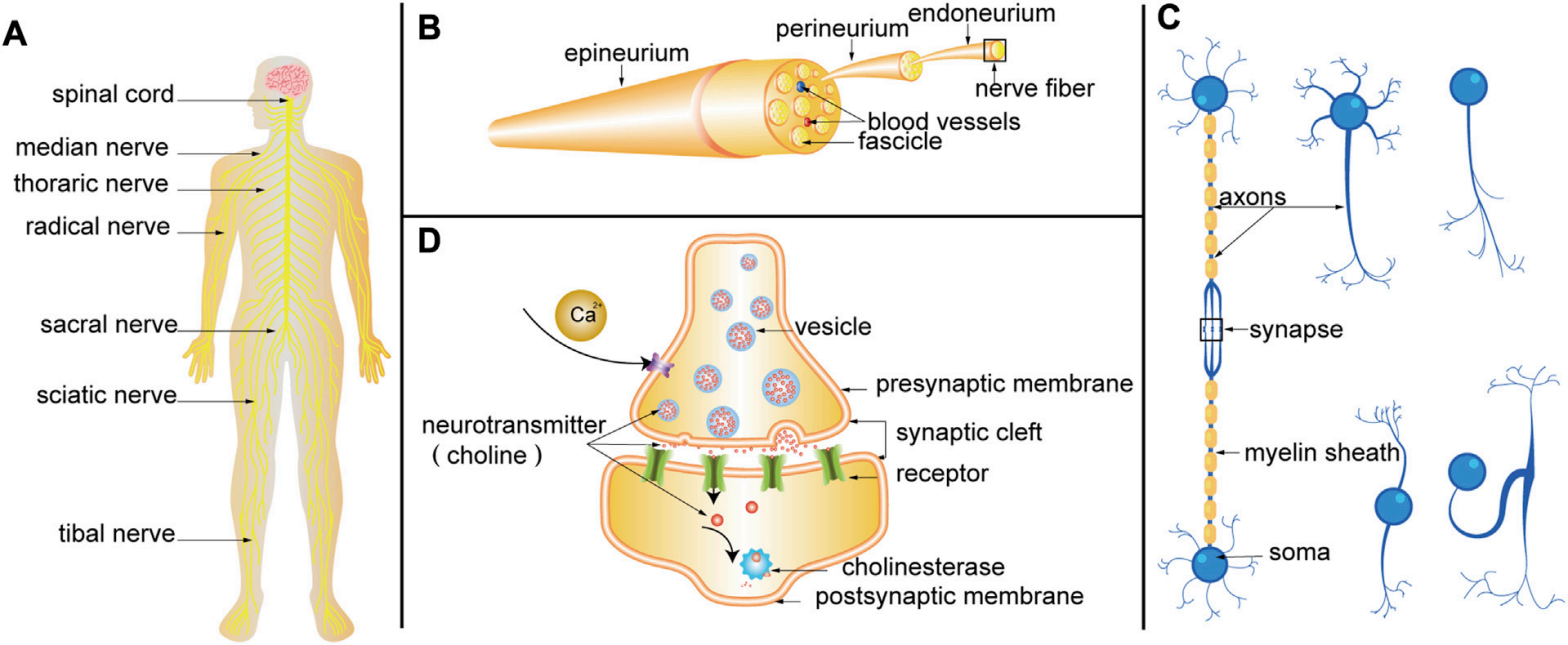
**(A)** Nervous system. **(B)** Schematic diagram of PN anatomy. **(C)** Structure of nerve fiber. Neurons are composed of somas and neurites. Neurites can be divided into axons and dendrites. Axons are coated with myelin to form nerve fibers. According to the number of neurites emitted by the somas, neurons can be divided into four categories: unipolar neurons, pseudo-unipolar neurons, bipolar neurons, and multipolar neurons. Axon terminals can form synapses with somas, dendrites, and axons. **(D)** Progress of synaptic transmission. When the nerve impulse is transmitted to the axon terminal, the permeability of the presynaptic membrane to calcium ions is increased, and a large amount of calcium ions enter the presynaptic membrane to promote the tight fusion of the vesicle and the presynaptic membrane, and the rupture opening occurs. The choline (a kind of neurotransmitter) in the vesicles is released into the synaptic cleft and reaches the postsynaptic membrane through diffusion, where they bind to receptors on the postsynaptic membrane, changes the permeability of the postsynaptic membrane to ions, and causes excitatory or inhibitory changes in the postsynaptic membrane. Finally, the choline is hydrolyzed by cholinesterase.

In the realm of optical molecular imaging technology, the efficacy of surgical procedures is heavily reliant on the use of fluorescent agents. Unlike the medical device market, clinical approval has only been granted for a select few fluorescent agents, namely, fluorescein (FS), methylene blue (MB), 5-aminolevulinic acid (5-ALA), and indocyanine green (ICG). These agents have been clinically approved for uses such as tumor labeling (Waqas et al., 2017), angiography (Chotard et al., 2019), ureterography (Kaplan-Marans et al., 2019), sentinel lymph node identification (Chandrappa et al., 2020; Zhang et al., 2022) and cholangiography (Pardo Aranda et al., 2023). Compared to tubular structures such as blood vessels, ureters, and bile ducts, the bundled structure of nerve fibers makes it challenging for fluorescent agents to color, thus *in vivo*, imaging is difficult to achieve (Figures 2A,B). This problem has led researchers to develop neural fluorescent agents, and the number of studies in this field has increased in recent years. Although preliminary results of these probes are promising, there is insufficient research on their drug metabolism, dosage, pre-injection time, and toxicity. The clinical use of fluorescent agents in nerve bioimaging is limited, and there are few relevant clinical reports.

This paper provides a systematic summary of clinical and preclinical studies on various fluorescent agents used in optical molecular imaging over the past two decades. The functions and characteristics of these agents in neural visualization under the guidance of optical molecular imaging technology are also described. The discussion includes a prospectus on the development prospects of optical molecular imaging technology-guided neural visualization, aimed at assisting researchers in related fields.

## 2 Neuro-nonspecific agents

Neuro-nonspecific agents are primarily utilized to enhance the visibility of nerves and their surrounding structures. This can be achieved through local injection or systemic injection, leveraging the physiological anatomy of the peripheral nerve (Samarasena et al., 2016). Furthermore, fluorescent agents can accumulate in peripheral nerve tissue through free diffusion and active reuptake (Schmued, 2016). By observing the timing and metabolic characteristics of fluorescent dyes in various tissues *in vivo*, we can effectively visualize nerve tissue intraoperatively (de Melo et al., 2016; Debie and Hernot, 2019; Walsh et al., 2019).

### 2.1 Neurovascular bundles agents

**FIGURE 3:**
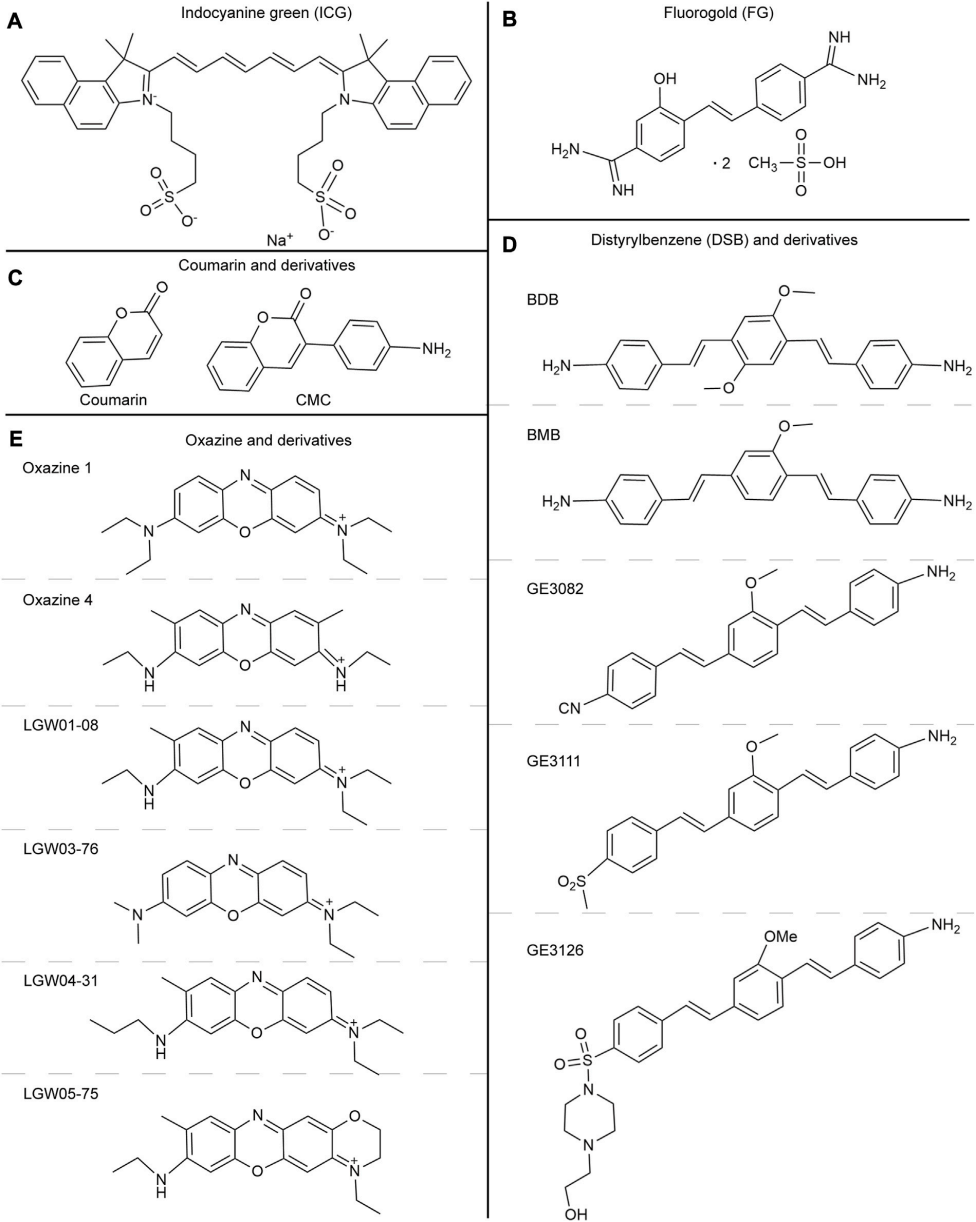
**(A)** The chemical structure of indocyanine green. **(B)** The chemical structure of Fluorogold. **(C)** The chemical structures of Coumarin and derivatives. **(D)** The chemical structures of Distyrylbenzene and derivatives (BDB, BMB, GE3082, GE3111, GE3126). **(E)** The chemical structures of Oxazine and derivatives (Oxazine 1, Oxazine 4, LGW01-08, LGW03-76, LGW04-31, and LGW05-75).

ICG (Figure 3A) is a water-soluble anionic, amphiphilic fluorophore, which is particularly attractive due to its excitation (λex = 778 nm) and emission (λem = 830 nm) (Zhou et al., 2016). ICG emits 800 nm ​​fluorescence and is the only NIR fluorescent agent approved by the FDA and the European Medicines Agency (EMA) for a small number of surgical indications (Giraudeau et al., 2014; Kaplan-Marans et al., 2019). Due to minimal toxicity, ICG has been utilized for many illness diagnoses and therapies under the direction of NIR fluorescence imaging, and this innovative intraoperative imaging technique is now employed to guide surgical accuracy (Chen et al., 2020). Neurovascular bundles are composed of interconnected nerve terminals and capillaries. Injecting ICG into a blood artery can identify the capillaries in the neurovascular bundle and detect the nerve indirectly by detecting blood flow (Jiang et al., 2015; Mangano et al., 2018).

The University of Arkansas Medical Department has successfully preserved the facial nerve and semicircular canal by utilizing fresh human head tissues through the systematic injection of ICG while under the guidance of NIR fluorescence imaging (Gragnaniello et al., 2010). At Taipei Veterans General Hospital, a standard dose of ICG diluted in 5 mL of sterile water was administered systemically into the accompanying vessels of the facial nerve canal to visualize the facial nerve after ossification based on the differences between cortical bone and the facial nerve during mastoidectomy. In 2014, facial nerve fluorescence imaging took a median duration of 32 s in 16 cases (Chen et al., 2015). The use of this technique may aid clinicians in accurately identifying the facial nerve or relevant anatomical landmarks, thereby reducing the incidence of complications related to facial nerve damage. However, due to the limited sample size and range of diagnoses, a standard protocol has yet to be established. Additionally, the study did not evaluate the post-surgical facial nerve function of patients, which presents significant limitations to the findings.

According to a study conducted by Peking University People’s Hospital, ICG fluorescence technology was found to accurately localize the thoracic sympathetic nerve. This result highlights the potential of ICG fluorescence technology in the field of medical imaging and diagnosis (Weng et al., 2016). Previous animal studies have suggested that the ideal protocol for thoracic sympathetic nerve fluorescence imaging before the surgery involves administering a dose of 5 mg/kg and allowing for a preparation period of 24 h before the operation. In clinical applications, ganglia have been observed from the stellate ganglion (SG) to the sixth thoracic ganglion (T6), along with the neuronal chain that connects them. Pathological findings have confirmed the progress of these studies (He et al., 2018). This study is the first to report fluorescence imaging of the sympathetic ganglia in individuals who did not experience adverse reactions to the medication.

In 2020, Kiyoshi Kanno and his team in Japan utilized indocyanine green (ICG) through systematic administration for NIR fluorescence imaging to improve the visualization of the anatomical connection between the lesion and pelvic autonomic nerves during the treatment of a severe case of pelvic endometriosis. The ICG revealed that the hypogastric nerve and plexus played a significant role. Fortunately, there were no postoperative complications related to pelvic autonomic nerve injury (Kanno et al., 2021). The feasibility and safety of utilizing NIR fluorescence imaging with ICG to detect pelvic nerves during radical hysterectomy for cervical cancer were examined in a clinical study conducted by the Obstetrics and gynecology department of Nanfang Hospital, Southern Medical University, China, from February 2020 to June 2021. The study found that in 63 cases, the bilateral identification rates of the obturator nerve (ON), the genital femoral nerve (GN), and the inferior ventral nerve (HN) were 100%, 93.7%, and 81.0%, respectively. The efficacy of intraoperative imaging was also validated by postoperative pathology data. This novel method for identifying pelvic nerves has the potential to reduce nerve damage and improve patients’ prognoses (He et al., 2022). Previous clinical investigations in this sector have shown that the optimal concentration of ICG is 4.5 mg/kg and that the injection should be administered 24 h before surgery to achieve the maximum SBR value (Jin et al., 2022). ICG’s affinity for different tissues varies, but it has a particularly low affinity for nerves. Therefore, a higher dosage of ICG is required to visualize nerves. Although the primary objective of these investigations was to evaluate the feasibility and safety of this approach, no randomized controlled trials were performed to compare it with traditional surgery. As a result, its efficacy must be further established.

ICG is highly lipophilic, which makes it readily accumulate in adipose tissue during clinical practice. Therefore, the SBR value may decrease if the injection time is not precisely measured. Despite its low quantum yield, poor optical stability, and lack of targeting, ICG has better optical tissue penetration than other colors. Therefore, it is believed that a combination of radioactive and ICG would be the most effective approach (Handgraaf et al., 2014; SAJI, 2017).

### 2.2 Axonal transport agents

Axonal transport (AT) is a fundamental cellular process that plays a critical role in the development and preservation of neuronal structures and connections (Tesco and Lomoio, 2022). The development of novel agents and histochemical methods has always been driven by specific needs. One such need was to develop agents that could be administered to specific brain regions *in vivo*, allowing the subsequent visualization of neuronal connectivity through the AT of the agent. Fluorescence imaging in cranial nerves (CN) or peripheral nerves (PN) can be achieved through the use of fluorescent agents such as Fast Blue (FB, λex = 365 nm and λem = 420 nm), Fluorogold (FG, λex = 361 nm and λem = 536 nm, Figure 3B), NeuroTrace (λex = 500 nm and λem = 525 nm), and Dio/FastDio (λex = 484 nm and λem = 501 nm), which rely on anterograde and retrograde axonal transport [Figure 2C (Schmued, 2016; Walsh et al., 2019)]. Due to its reliance on AT mode of operation, local injection is exclusively available for users.

Fast Blue is a widely used agent in preclinical models. Recent studies have demonstrated that injecting FB into various nerves, such as thoracic spinal nerves, cerebral cortical nerves, thoracolumbar sympathetic nerves, and facial nerves, can produce exceptional imaging results for nerve development and lesion localization when exposed to UV light (Anderson et al., 2013; Schmued, 2016). Advantages of this agent include brightness, sensitivity, contrast, stability, persistence, and compatibility with various labeling studies. This agent can be used to locate afferent or efferent connections within regions of interest in the brain. This discovery opens up new avenues for investigating the control mechanisms of both the neurological and endocrine systems, as well as for developing treatments for nerve injuries.

FG is widely considered the most reliable method for preclinical retrograde neuronal labeling due to its bright fluorescence, efficient labeling, lack of diffusion to neighboring cells, ability to label cell body cytoplasm and dendrites, and resistance to fading, suitable for targeted deep imaging of the nervous system (Schmued, 2016). Its visibility under ultraviolet light has been extensively utilized in animal research that involves brainstem-related injuries (Barham et al., 2022). Through experimentation, it was discovered that FG’s two-photon excitation spectrum ranges from 720 to 990 nm. Among the wavelengths within the microscope’s tuning range, 720 nm is the ideal two-photon excitation wavelength, while 760 nm is the highest lifetime contrast wavelength (Miller et al., 2021; Sotoudeh et al., 2022). The broad fluorescence emission spectrum of FG often results in spectral overlap with other fluorophores, which can impede multicolor imaging. Meanwhile, FG is known to be toxic to both sensory and motor neurons, resulting in reversible motor and sensory deficits (Mi et al., 2019). This may become an important reason to hinder its clinical application.

NeuroTrace, which is currently the only marker for mouse brain pericytes, was initially utilized to identify neurons in preserved brain tissue (Damisah et al., 2017). Upon introducing the fluorescent agent to a primary culture of human pericytes, green fluorescence was observed (Kim et al., 2022). In the study of gastrointestinal motility disease, researchers utilized a confocal laser microendoscope to observe neuronal cells and neural fiber networks in the submucosa and muscle lumina without the need for invasive mucosal resection by activating NeuroTrace (Samarasena et al., 2015; Samarasena et al., 2016). In the mouse brain, NeuroTrace did not enter either neurons or glial cells, indicating that its transport system likely involves molecules expressed by human and rodent pericytes. Therefore, NeuroTrace has been identified as a valuable marker for pericytes, and further research on the molecules involved in its transport to pericytes will likely enhance its utility. These findings will contribute to the future development of pericyte-specific antibodies.

In this study, Dio/FastDio and other carbon flower fluorescent agents were originally used for cell labeling. The aim was to identify the facial nerve in animal models of head and neck tumor resection using a fluorescent anatomic microscope. The results showed that carbon flower fluorescent agents had a strong fluorescence effect and that neurotoxicity was only apparent in cases of temporary paralysis (Dogru et al., 2008). The use of FastDio also made the facial nerves detectable under white light illumination (de Melo et al., 2016). This agent labeling is compatible with nerve survival and function. Such strategies may be particularly helpful in the management of benign tumors that splay and distort the facial nerve, such as vestibular schwannomas or large parotid pleomorphic adenomas. They may also prove useful in confirming nerve entry zones into the central nervous system, as in the cochlear nucleus, to facilitate optimal placement of auditory brain stem implants.

In practical applications, it is crucial to select suitable imaging equipment and imaging conditions due to the varying physical and chemical properties of these fluorescent agents. When selecting a fluorescent agent, it is important to consider its characteristics such as fluorescence intensity, tissue penetration, imaging depth, and wavelength comprehensively. These factors should be taken into account to make an informed decision. These agents have various characteristics including high safety and wide applicability. They are used to detect apoptotic, necrotic, and degenerated neurons resulting from different types of injuries, such as physical trauma, neurodegenerative diseases, and various neurotoxins. However, their development outcomes have been disappointing, and their lengthy pre-injection period in experiments may increase surgical risks in future clinical applications. Additionally, staining is dependent on AT, limiting its use to local injection. Due to these factors, neuro-nonspecific agents have not been widely accepted.

## 3 Neuro-specific agents

The discovery of multiple nerve marker binding sites has enabled the use of fluorescence imaging *in vivo* to quickly locate nerves in targeted areas, thereby significantly reducing the risk of nerve damage during surgery. Different fluorescent agents are utilized for various therapeutic applications, owing to their unique modes of action, binding locations, and methods of administration. (Davila et al., 2008). Neuro-specific agents are classified into distinct categories based on the specific target binding sites they bind to.

### 3.1 Myelin-specific targeted agents

Myelin is a multilayer membrane structure surrounding the axons of neurons, mainly composed of lipids and myelin proteins necessary for normal neuronal function. Central myelin and peripheral myelin are composed of several proteins, proteolipid protein (PLP), myelin-associated glycoprotein (MAG), protein P0, chondroitin-sulfate proteoglycans, reticulon-4 (Rtn-4)/NogoA, all of which may be candidate targets with different affinities for fluorescent agents (Savaskan et al., 2009), but its exact mechanism is currently unclear.

#### 3.1.1 Coumarin and derivatives

Coumarins are important fluorescent compounds, with some derivatives being used as fluorescent agents for nerve labeling during surgery (Lee et al., 2006; Lin et al., 2014). In 2010, a coumarin derivative called 3-(4-aminophenyl)-2H-chromen-2-one (CMC) was developed to selectively label myelinated nerve fibers in a mouse model (Wang et al., 2010). Since then, a novel coumarin derivative called fluorinated imaging compound (FIC) has been tested and proven to possess more neuro-binding properties than CMC (Lin et al., 2014). In these studies, researchers have demonstrated that the lipophilicity of fluorescent agents plays a crucial role in their ability to permeate the brain and bind to myelin. When considering the combination of fluorescent agents and myelin, it is important to take into account not only the similarity in lipophilicity but also other relevant physical and chemical properties. For example, the presence of hydrophilic groups, such as hydroxyl groups, in a hydrophobic environment can decrease binding affinity. In contrast, the lower electron density of nitrogen can enhance binding affinity. Coumarin and derivatives (Figure 3C) can be injected intravenously and selectively stain the medullated nerves along with blood flow. This property allows for the identification of medullated nerves in different regions. Fortunately, coumarin derivatives have low toxicity, making them viable options for myelin fluorescent agents in clinical imaging modalities and translational research.

#### 3.1.2 Distyrylbenzene and derivatives

**FIGURE 4:**
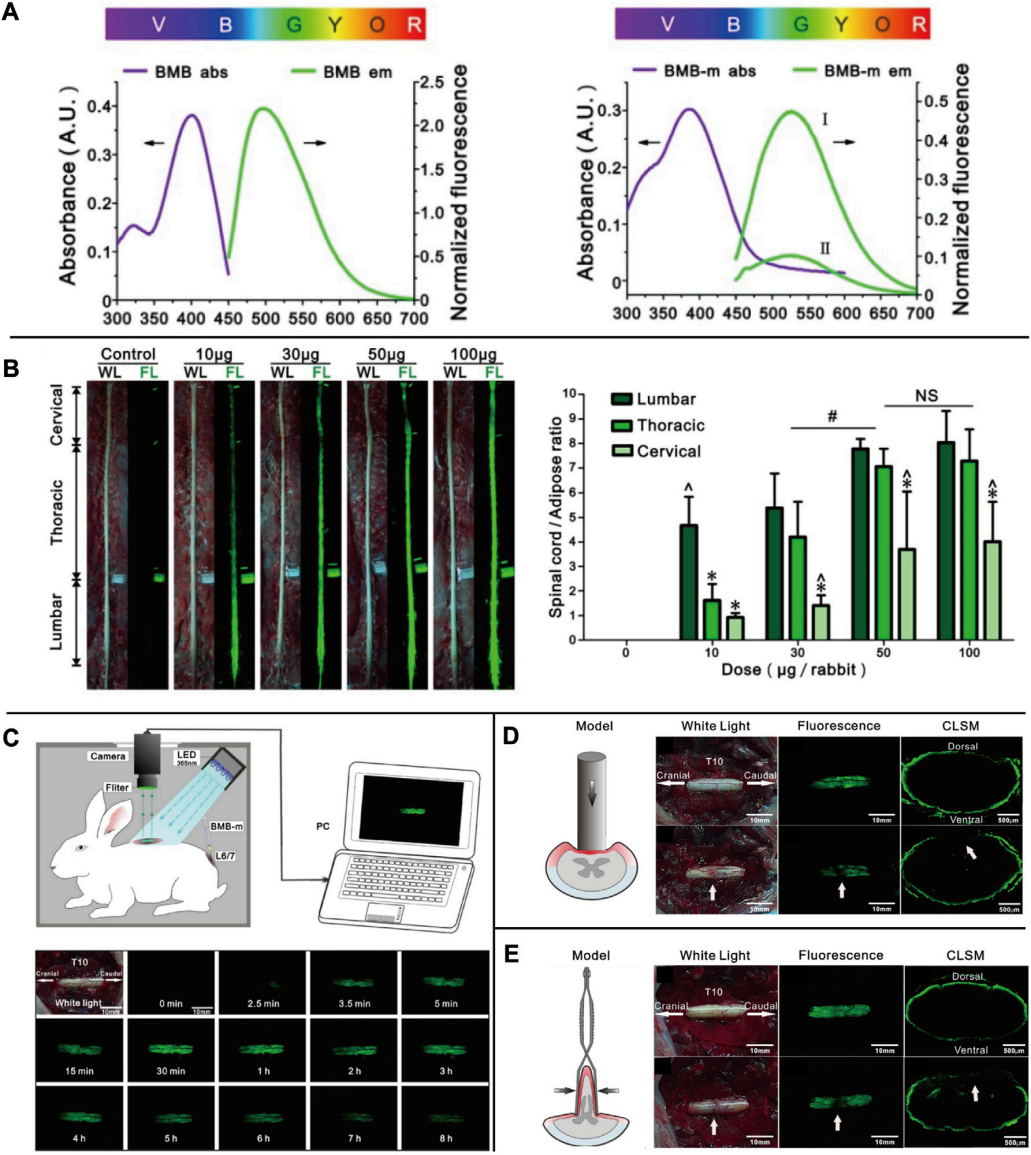
**(A)** The UV absorbance and fluorescence emission spectra of BMB and BMB-m. **(B)** Dose-response of BMB imaging of the spinal cord at 30 min post-injection. Observation of the spinal cord under white or UV light after epidural administration of BMB-m solution at different BMB doses (from 10 to 100 μg/rabbit) and the fluorescence ratios of the spinal cord to adipose (SC/A) at lumbar, thoracic, and cervical spinal cord segments at different doses. **(C)** Schematic illustration of real-time and *in situ* monitoring of the fluorescence at the T10 spinal cord after a single dose of epidural BMB (50 μg, in BMB-m formulation) at L6/7 and the represent fluorescence images of the T10 spinal cord at different time points. **(D)** Detection of iatrogenic spinal cord injury by BMB imaging. *In situ* images of T10 spinal cord under white light before, UV light, and pathological section under UV light after acute injury with Rivlin method. **(E)** Detection of spinal cord injury by BMB imaging. *In situ* images of the T10 spinal cord under white light before, UV light, and pathological section under UV light after acute. *Copyright 2017, Ivyspring International Publisher.*

**FIGURE 5:**
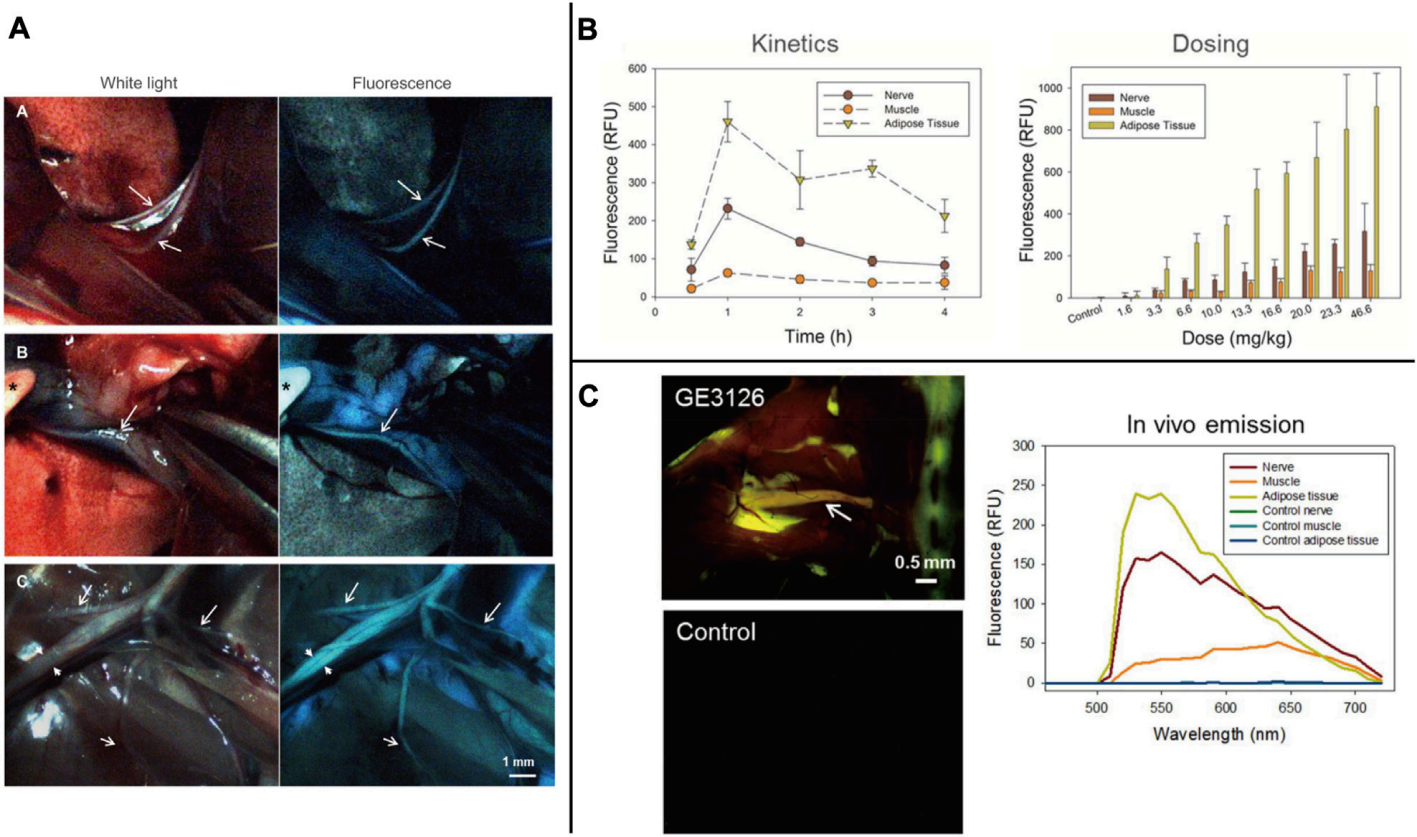
**(A)** Individual frames extracted from real-time video recorded during surgery of a rat injected with 12.2 mg/kg of GE3126 using a custom dual-mode laparoscopic imaging instrument. **(B)** The research of GE3126 for fluorescence imaging of mouse sciatic nerve in kinetic and dose. **(C)** Fluorescence multispectral image and emission spectra of different tissue. *Copyright 2015, Public Library of Science.*

Autologous fluorescent compounds such as distyrylbenzene (DSB) fluorescent agents have varying emission wavelengths in the NIR range, enabling differentiation between adipose and brain tissues through optical signals. Further studies have shown that these compounds can be used to visualize neuronal tissues in mice, rats, and pigs intravenously (Gibbs et al., 2013). To study illnesses of the central nervous system caused by myelin lesions, scientists have developed (E, E)-1, 4-bis(4′-aminostyryl)-2, 5-dimethoxy-benzene (BDB). This compound is highly selective for myelin and allows for the identification of demyelinating lesions *in vivo* through animal testing (Wu et al., 2006). The BDB research has provided a new method for identifying myelin *in vivo*. The permeability of the blood-brain barrier can be determined by examining lipophilicity, which is a critical indicator. In DSB, another molecular probe called (E, E)-1, 4-bis (p-aminobenzene)-2-methoxy-benzene (BMB) is used, which is highly lipophilic. When BMB is encapsulated in a micelle (BMB-M) and injected epidurally, it can easily cross the blood-brain barrier and mix with the cerebrospinal fluid (CSF). As a result, the spinal cord’s peripheral area of the white matter can be rapidly stained [Figure 4 (Waterhouse, 2003; Liu et al., 2017)]. This technology allows for real-time monitoring of the spinal cord during surgery, reducing the risk of postoperative spinal cord damage. BDB and BMB, which can be radiolabeled with 11C both *in vitro* and *in vivo*, have the potential to serve as *in vivo* myelin fluorescent agents in clinical practice. Their ability to permeate the blood-brain barrier makes them suitable for use with PET-CT to conduct myelin imaging (Stankoff et al., 2006; Wu et al., 2008). A set of DSB molecular probes are currently being developed. The Tan Hehir group’s myelin-targeting fluorescent agent, GE3111, is being utilized to view nerves in mice by using a fluorescent device with a wavelength of 405 nm and light intensity below 2.5 mW/cm2. It was found that GE3111 has improved water solubility and lower lipophilicity when compared to the group’s previous formulation, GE3082 (Gibbs-Strauss et al., 2011; Cotero et al., 2012). After conducting further research, the team utilized GE3126, a small molecule fluorophore that is compatible with a dual-mode laparoscopic imaging instrument capable of both color and fluorescence imaging. This fluorophore has improved water solubility and pharmacokinetics while also reducing lipophilicity. Additionally, it effectively eliminates fluorescence interference from non-specific adipose tissue [Figure 5 (Cotero et al., 2015)]. By examining the physical and chemical properties of DSB and derivatives (Figure 3D), we discovered that despite their abundance of double bonds, the symmetry of these molecules results in the absorption and emission of ultraviolet and visible light only due to the molecular symmetry, while failing to achieve absorption and emission in the near-infrared region. Even though it presents a significant challenge, this basic structure is expected to serve as a foundation for the development of new fluorescent agents that specifically target nerves in the future. This approach offers immediate benefits for both patients and surgeons in surgical operations involving nerve identification.

The common feature of these fluorescent substances is their ability to pass through the blood-nerve barrier (BNB). Therefore, relevant animal experiments are typically conducted through intravenous administration. Due to their high lipophilicity, identifying most of these molecular groups from the surrounding adipose tissue and other non-neural tissues is difficult. Therefore, lipophilicity should be considered a significant factor when selecting fluorescent agents. Although there are no published studies on their cytotoxicity and pharmacokinetics, the majority of these medications can cross the blood-brain barrier. In addition, fundamental experimental research on solvent selection in intravenous formulations is lacking.

### 3.2 Axon-specific targeted agents

#### 3.2.1 Neurophagic viruses

Neurophagic viruses have been demonstrated to target the nervous system by infecting cells or tissues (Glasgow et al., 2016; Schubert et al., 2018). As demonstrated by the use of rabies virus, herpes simplex virus, and adenovirus. These viruses can be modified to produce lentiviral vectors that attach to carrier proteins and retrogradely transport the virus to brain tissue, allowing for specific targeting (Cronin et al., 2005; Chen et al., 2020). The heavy chain of the tetanus toxin can enhance immunity and can be combined with trisialoganglioside GT1b to produce fluorescent markers that can be used for staining neurons (Toivonen et al., 2010; Yousefi et al., 2014). Despite progress in research, utilizing engineered viruses that express specific reporter genes and recombinant viruses that express green fluorescent proteins to track nerves has proven difficult due to the viruses’ pathogenicity and infectiousness, making their clinical application challenging. Detection of nerve infection in both time and space is possible (Enquist and Card, 2003). Regarding its pathogenicity and infectivity, there is a potential future possibility of integrating the virus with magnetic nanoparticles. This integration could involve introducing a specific magnetic field *in vitro* to concentrate the virus at a targeted location. Additionally, applying a biodegradable polymer on the virus surface could help reduce its toxicity and enhance its breakdown within the body.

#### 3.2.2 Non-virus agents

Non-virus-targeted agents are highly valued for research due to their safety and accessibility (Chen et al., 2020). For example, earlier research has demonstrated the effectiveness of several anti-ganglioside monoclonal antibodies (MABS) as selective nerve delivery vehicles for peripheral nerve fluorescence imaging (Massaad et al., 2015). In this study, researchers combined biotinylated dextran amines (BDA) with red fluorescent carbonized polymer dots (CPDS) on brain tissues to create a new family of nanoscale neuro-fluorescence agents called BDA-CPDs. These agents demonstrated high biocompatibility and minimal toxicity both *in vitro* and *in vivo* (Liu et al., 2019). In recent years, there has been a concerted effort to develop small molecule and peptide complexes that can effectively target the neuronal signal transduction pathway. In animal studies, researchers have utilized Hsla-FL, which is produced by combining Hs1a, a substance that acts on the sodium channel, with the Cy7.5 fluorescent agent. Additionally, BChE-IRagent has been created by labeling butyrylcholinesterase (BChE), which acts on choline (Figure 2D), with NIR fluorescent agents (Johnson et al., 2009; Gonzales et al., 2020). Further research is required to enhance the intraoperative use of these synthetic compounds. The agents mentioned above are composed of fluorescent dyes and special molecules. These special molecules can influence the generation of electrical activity in the nervous system and bind specifically to it. These agents possess excellent tissue penetration ability and photostability, with minimal adverse effects on the body. As a result, they are considered ideal neuro-specific agents. These researches present new ideas for the future development of agents, with a focus on exploring the electrophysiological activities of nerves.

### 3.3 Neurilemma-specific targeted agents

The connective tissue membranes surrounding nerves are composed of the endoneurium, epineurium, and perineurium. These membranes contain a high concentration of proteoglycans (PGs), which are the most prevalent proteins in the nerve fiber connective tissue (Dyck and Karimi-Abdolrezaee, 2015). These proteoglycans (PGs) are composed of a protein core with attached glycan chains (Ruoslahti, 1988), resulting in five different types of PGs: chondroitin sulfate proteoglycan (CSPG), heparan sulfate proteoglycan (HSPG), keratan sulfate proteoglycan (KSPG), dermatan sulfate proteoglycan (DSPG), and hyaluronan proteoglycan (HP) (Scott, 1988; Reitsma et al., 2007).

#### 3.3.1 Lectins

**FIGURE 6:**
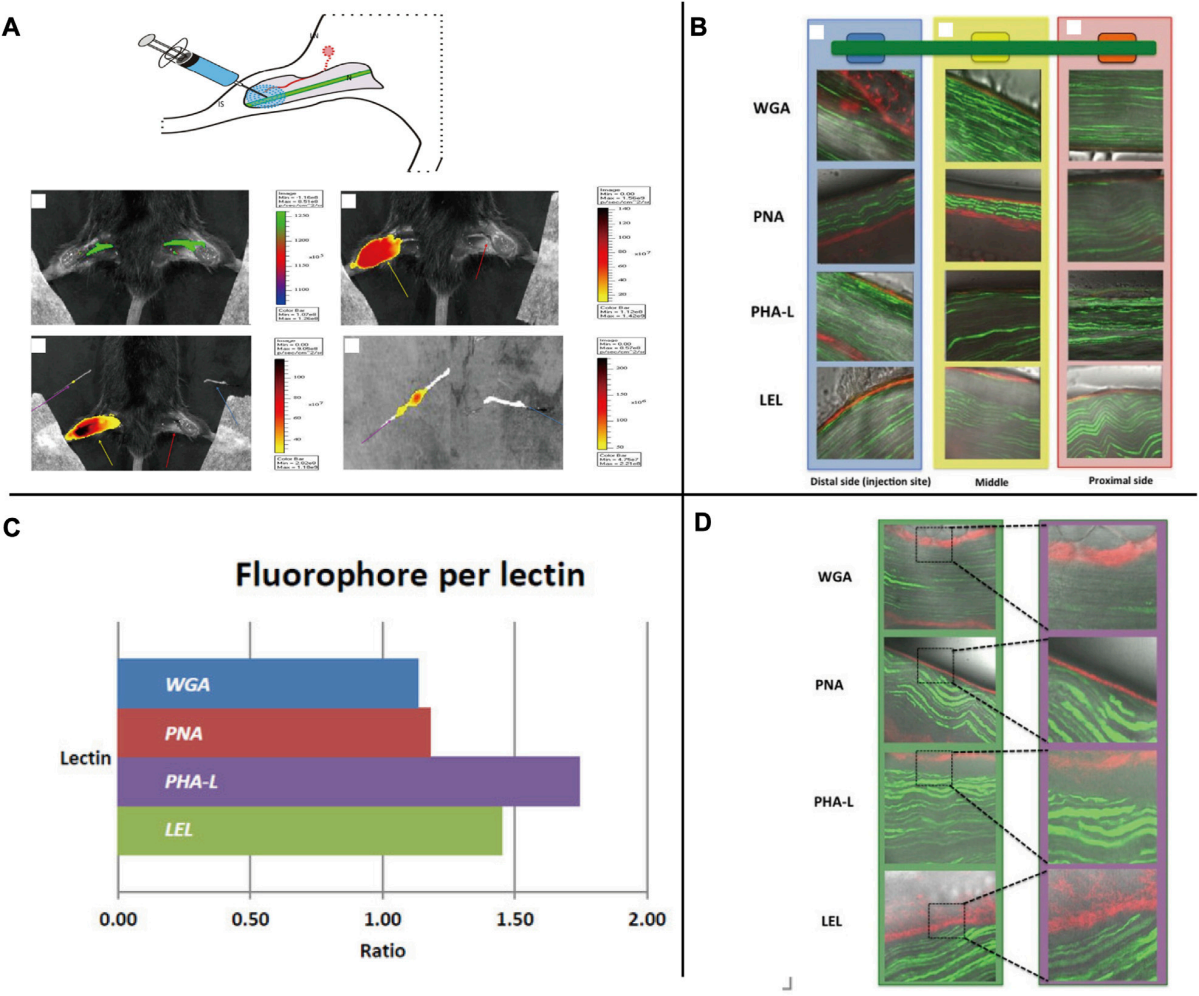
**(A)** Schematic representation of the local injection of Cy5-lectins and fluorescence imaging of mouse sciatic nerve. **(B)** Binding mode after *in vivo* local administration. The fluorescence signal in nerves from Thy-1 YFP mice (YFP in green and Cy5 in red) was traced from the injection site to the middle and the proximal side of the nerve. In all cases, staining of the epineurium was observed with a decrease in signal when the distance from the injection site increased. **(C)** Cy5/Lectin labeling ratio per lectin. **(D)** *Ex vivo* incubation confirmed the *in vivo* localization of staining. (YFP in green and Cy5 in red). *Copyright 2014, MDPI.*

Lectins are a group of proteins ranging in size from 4–10 nm, they can bind to specific sugar groups, known as oligosaccharides, which leads to an affinity with proteoglycans (PGs) found on the extracellular matrix of peripheral nerve tissue (Dyck and Karimi-Abdolrezaee, 2015). In 2014, a team led by Fijs Willem Bernhard van Leeuwen in the Netherlands utilized Cy5 to combine four lectins - wheat germ agglutinin (WGA), peanut agglutinin (PNA), red kidney bean agglutinin (PHA-L), and tomato lectin (LEL) - for a fluorescence experiment on mouse sciatic nerve. The lectins were locally injected and resulted in SBR values of 1.86, 1.26, and 1.12 for WGA, LEL, and PHA-L, respectively, with the background muscle serving as the control [Figure 6 (Kleinjan et al., 2014)]. Lectins have a high potential for therapeutic application due to their exclusive staining of the epineurium and minimal systemic toxicity. However, their use is limited as they can only be administered through local injection and may not penetrate deep nerve tissue. Furthermore, their interaction with surrounding connective tissue may interfere with imaging.

#### 3.3.2 Oxazine and derivatives

**FIGURE 7:**
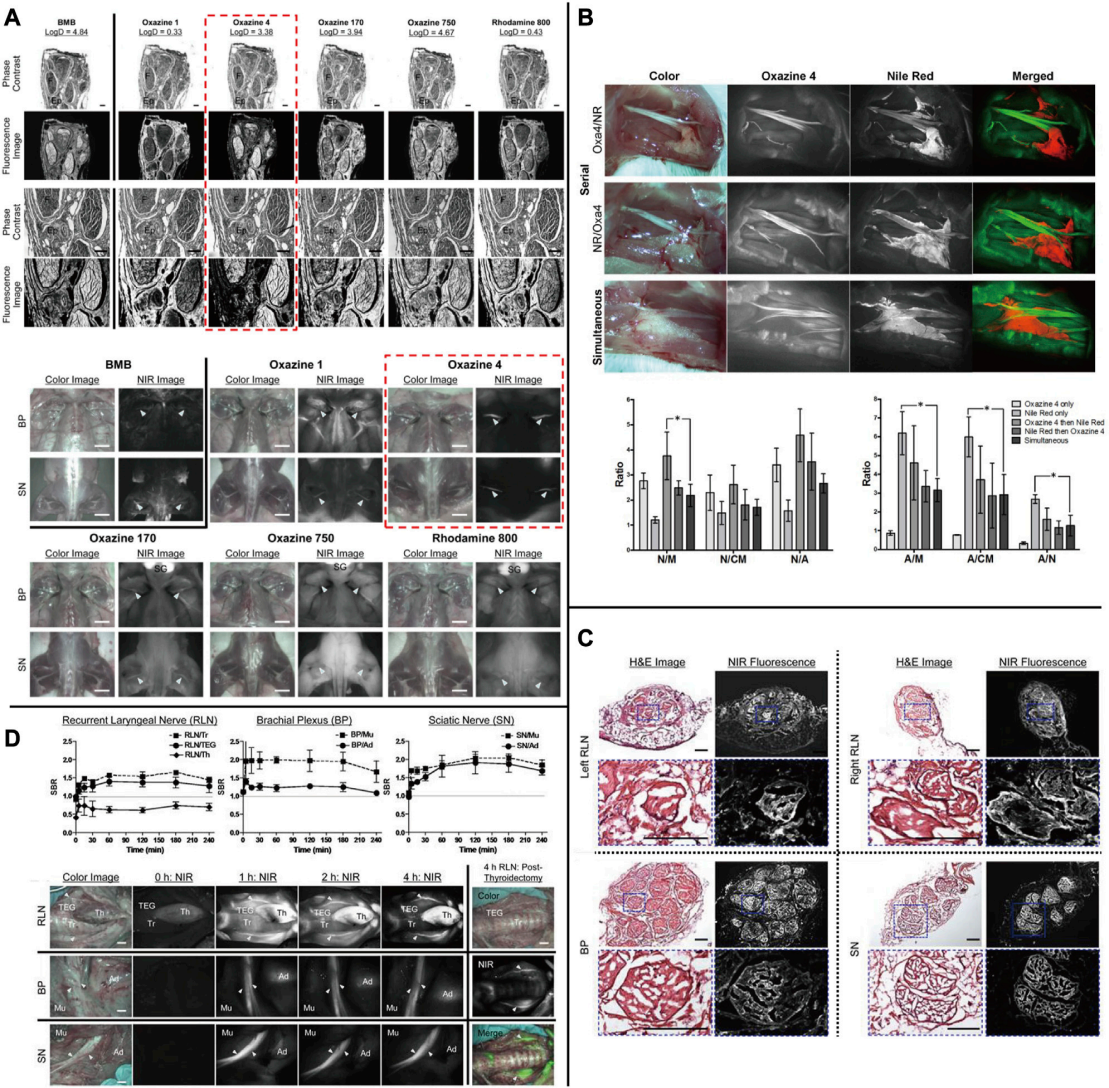
**(A)** Chemical structure of nerve-targeting fluorophores and the *Ex Vivo* and *in Vivo* screening assay of fluorophores for nerve-specificity with them. **(B)** Dual fluorophore staining technique for nerve and adipose spectral separation with Oxazine4 and Nile Red. **(C)** Fluorescence microscopic images of the laryngeal nerve (RLN), brachial plexus (BP), and sciatic nerve (SN) with Oxazine4. **(D)** Quantitative time-course assessment of SBR (Mean ± SD) for recurrent, and real-time intraoperative fluorescence images of RLN (top), BP (middle), and SN (bottom) acquired at different time points (*T* = 0, 1, 2, and 4 h). *Copyright, Ivyspring International Publisher.*

The oxazine scaffold, which currently emits at visible wavelengths with nerve-specific derivatives, presents a potentially promising and flexible architecture. Directed oxazine synthetic engineering holds promise in the identification of translational small-molecule nerve contrast candidates that can effectively delineate nerves in intricate surgical settings. Gibbs and his team constructed a library of neuro-specific fluorescent agents using oxazine-1 and oxazine-4 as scaffolds. They evaluated four oxazine compounds (LGW01-08, LGW03-76, LGW04-31, and LGW05-75) through an *in vivo* neuro-fluorescence test ((Figure 3E) Wang et al., 2020). Compared to other groups in the library, oxazine 4 has demonstrated a more noteworthy experimental impact. A single intravenous injection of 0.25 mg/kg of oxazine 4 can provide nerve-targeting signals in the brachial plexus and sciatic nerve for up to 12 h. Additionally, oxazine 4 has successfully identified and highlighted the recurrent laryngeal nerve in pigs in real-time. However, oxazine 1 lacks neuronal specificity, despite its structural similarity to oxazine 4 (Figures 7A,C,D (Park et al., 2014; Barth and Gibbs, 2018)). In 2017, this group utilized a dual-color imaging technique to separate nerve and adipose tissue fluorescence through local injection by utilizing the differing spectral features of oxazine 4 and Nile red, a lipid-specific oxazine fluorescent agent [Figure 7B (Barth and Gibbs, 2017)]. In 2019, researchers compared the fluorescence intensity of oxazine 4 and rhodamine in nerve tissue to investigate the effect of distribution coefficient (log D) and total charge on nerve tissue targeting and quick off-target tissue clearance (Wang et al., 2016). Oxazine 4 has a low molecular weight and a logD of 3.38, allowing it to pass through the blood-brain barrier in a normal physiological environment. When stimulated with NIR light, it can make certain nerves visible that are not visible under white light, even in deep tissues. While animal experiments have shown promising results, its optical characteristics are not in the NIR range, making its clinical application prospects lower than those of coumarin and DSB derivatives. Additional neuro-specific fluorescent agents from the oxazine library with nerve-binding specificity could be considered potential clinical candidates.

#### 3.3.3 Nerve peptides

**FIGURE 8:**
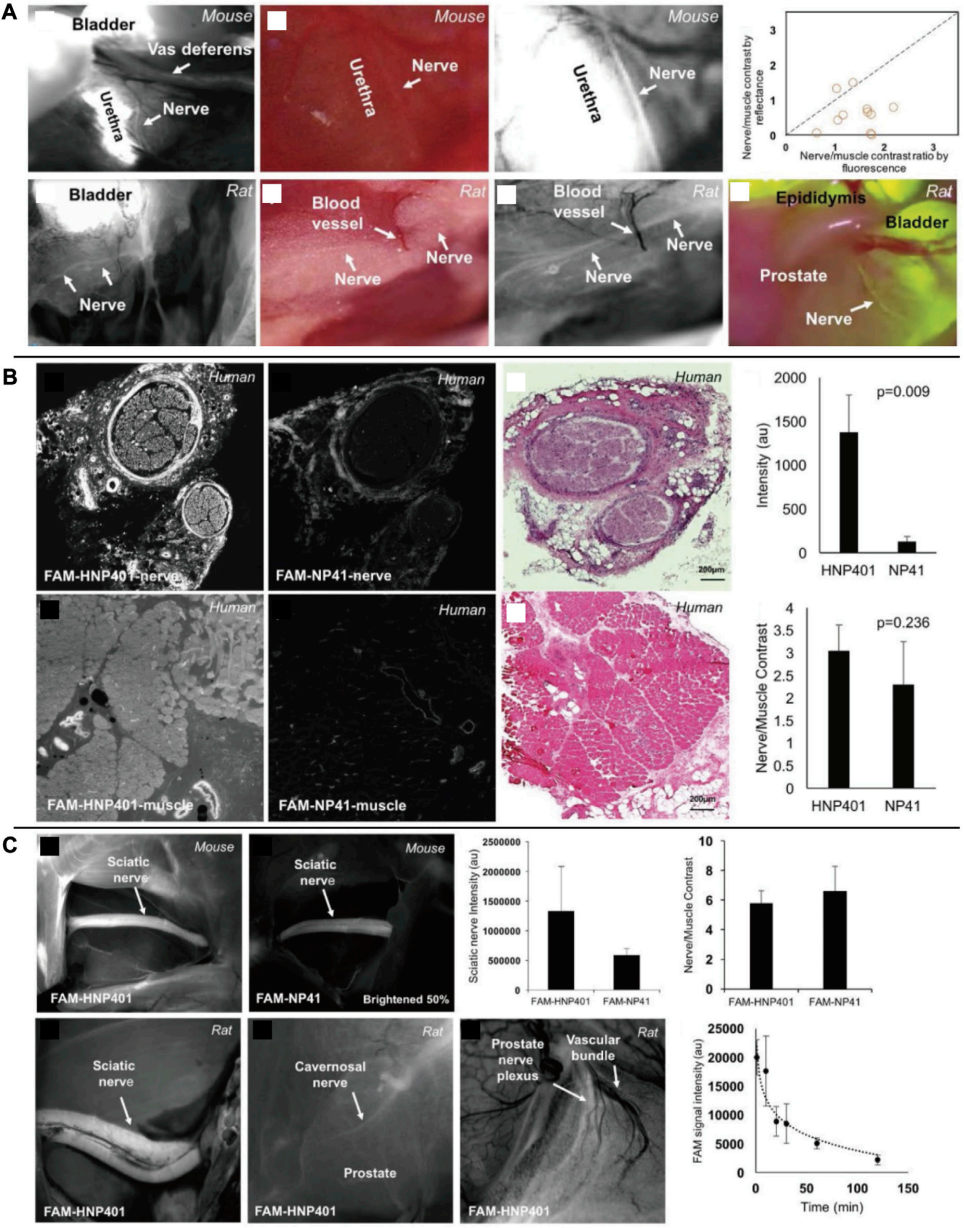
**(A)** *In vivo* fluorescent labeling of autonomic nerves in rodents. **(B)** Comparison of FAM-HNP401 and FAM-NP41 in binding and labeling of the human sural nerve. **(C)** *In vivo* imaging of nerve-binding peptides in mice and rats with pharmacokinetics. *Copyright 2018, Ivyspring International Publisher.*

Nguyen’s group has created a nerve peptide called NP41 through phage display. This peptide has shown the ability to selectively bind to almost all motor and sensory nerves in animal studies by binding to the fluorescein-5 (6)-carbonyl group (FAM) or Cy5. It has an SBR of 6.7 for sciatic nerves in the presence of peripheral muscles (Whitney et al., 2011; Wu et al., 2011). In trials using this peptide to identify nerve regeneration after damage, the time required for neural identification was reduced by 40% (Hussain et al., 2015). According to photo-oxidation analysis, laminin may be the binding target site (Glasgow et al., 2016). In a follow-up study, the researchers observed that nerve peptide 401 (NP401) had a stronger binding affinity for the peripheral nervous system. This result was demonstrated through visualization of prostatic nerves in mice and rats, as well as through greater contrast between nerve and muscle specimens in certain human tissues *in vitro* [Figure 8 (Hingorani et al., 2018)]. Protecting nerves is a critical clinical requirement for neuroimaging techniques. An interesting characteristic of these two neuropeptide sequences, in comparison to lipophilic dyes, is that they can function without the presence of myelin. This advantage sets them apart from other agents that specifically bind to myelin and axons. In addition to carrying Florescent agents to highlight nerves for image-guided surgery, the peptides could also function as a drug carrier. This combination system has the potential to promote the repair and regeneration of damaged nerves in the peripheral nervous system and spinal cord.

## 4 Summary and prospect

In the past decades, surgeons have adopted various diagnosis and treatment techniques and auxiliary methods. Among these, CT, MRI, United States, and nuclear medicine imaging have become common tools for preoperative diagnosis. These non-invasive imaging techniques help surgeons in planning surgery and improving treatment. However, some questions regarding surgical guidance and decision-making remain unanswered. Although United States, CT, and MRI are widely used, they have limited sensitivity and accuracy when it comes to detecting subtle lesions, and they are not suitable for real-time intraoperative feedback. United States is a valuable tool for diagnosing tumor diseases that have well-defined contours and clear boundaries in pathology, especially for PNS tumors (Misgeld and Kerschensteiner, 2006). However, it is important to note that high-resolution ultrasound relies on a network of hypoechoic bands and hyperechoic lines to identify nerves, which limits its ability to visualize deep tissue and hyperechoic structures of nerves, the United States can only detect approximately 30% of the total number of nerve bundles currently (Ohana et al., 2014; Carvalho et al., 2019). Optical molecular imaging technology utilizes the properties of fluorescent substances or externally introduced fluorescent compounds to aid physicians in making medical decisions. This is achieved by capturing optical signals using specific optical imaging equipment (Nishio et al., 2019; Liu et al., 2021). Compared to the United States, optical molecular imaging technology offers notable advantages in terms of imaging depth and clarity. In addition, optical molecular imaging technology can potentially visualize the parts that the fluorescent agent can reach during the operation. Recently, researchers have proposed a multimodal tumor diagnosis strategy by combining MRI and fluorescence imaging systems (McCann et al., 2009; Reynders et al., 2021; Song et al., 2021). This approach has demonstrated unique advantages in the multiple imaging of target tissues *in vivo*. The strategy leverages MRI to obtain accurate tumor volumes and utilizes fluorescent probes to measure tumor function and protease activity responses. This enables the quantification and tracking of tumor histological changes in a non-invasive manner. In molecular imaging of nuclear medicine, the imaging effect is closely related to the nuclear properties of radioisotopes, such as decay type, photon energy, and half-life. The use of foreign radioactive elements may cause adverse reactions in the body. In the field of molecular imaging, the effectiveness of nuclear agents is closely related to the nuclear properties of the radioisotopes used, including decay type, photon energy, and half-life. However, the use of radioactive elements can lead to negative bodily reactions.

During surgery, the surgeon relies on both touch and vision to make decisions. However, the rise of laparoscopic surgery, which is minimally invasive, has led to a decrease in the surgeon’s tactile sense. As a result, surgeons now depend heavily on intraoperative images. Fortunately, optical molecular imaging technology has advanced significantly in recent decades. As it continues to be integrated and improved upon in surgical procedures, it has paved the way for fluorescence-guided surgery, molecular image-guided surgery, and targeted surgery. These new directions have the potential to lead to further advancements in surgical techniques (Laguna, 2014; Benson et al., 2021; Lauwerends et al., 2021; Chand and Polom, 2022). Optical molecular imaging technology offers safe and convenient means to achieve high spatial and temporal resolution. This technology is useful for clinical diagnosis and analyzing biological phenomena, monitoring molecular content, observing the distribution of target tissues, and visualizing different biological tissues. It has played an irreplaceable role in revealing disease occurrence, development, and treatment and has opened up new avenues for research (SAJI, 2017; Wu et al., 2021). The advancement of this technology has resulted in the creation of various technologies including narrow-band imaging (Herr, 2014), photodynamic diagnosis (Didamson and Abrahamse, 2021), optical coherence tomography (Wang et al., 2022), and confocal laser endoscopic imaging (Pilonis et al., 2022).

The NIR fluorescence imaging system is continuously improving with the increasing utilization of various fluorescent agents in clinical applications (van Willigen et al., 2017). The researchers discovered that the fluorescent agent’s excited or emitted light could be absorbed by various cellular structures, including water, hemoglobin, oxygenated hemoglobin, skin, fat, and proteins. This absorption would significantly hinder the technology’s usability (Hemmer et al., 2016; Zhao et al., 2018). The spatial resolution of fluorescence systems is significantly reduced due to biological tissues’ strong optical scattering and absorption properties. This problem creates a significant challenge in achieving maximum imaging depth of biological tissues. In addition, the background signal produced by body tissues, known as spontaneous fluorescence, can decrease the SBR and lower the quality of the resulting image. However, adjusting parameters such as excitation duration and frequency harmonics can improve the imaging effect and mitigate the effects of scatter, ultimately resolving this issue (Chakraborty et al., 2019). In comparison to NIR-I, NIR-II fluorescence imaging offers advantages such as reduced tissue scattering and weaker tissue spontaneous fluorescence resulting in deeper penetration and higher spatial resolution in biological tissues, which can minimize background interference. However, the development of NIR-II fluorescent agents has been hindered by challenges such as poor water solubility, stability, fluorescence efficiency, biocompatibility, and metabolic rate (Frangioni, 2003; Miao et al., 2018; Liu et al., 2019). Researchers have been searching for fluorescent materials, such as probes and agents, that possess two critical characteristics at least. Firstly, these materials must effectively overcome the spontaneous fluorescence caused by thick and opaque tissue scattering to ensure the clearest possible images (Feng et al., 2013; Cao et al., 2018). Secondly, it must possess good biological stability and ideally cluster in the target tissue to produce fluorescence images that are specific to the target tissue (Feng et al., 2013).

Based on the above characteristics, researchers have become interested in quantum dots (QDs), which are inorganic semiconductor nanomaterials developed using certain metal elements like Cu, Cd, and Pb. QDs consist of a fluorescent core and a semiconductor shell. The surface of the shell is modified with specific targeting groups and other biomolecules using bio-coupling active targeting methods (Zhao et al., 2018; Yu et al., 2019). Numerous studies have confirmed that NIR QDs hold immense potential in preclinical research due to their superior brightness, sensitivity, specificity, and optical stability when compared to other fluorescent agents (Gil et al., 2021). The potential toxicity of NIR QDs in biological systems is a major concern for researchers. If the chemical stability of quantum dots *in vivo* is compromised, it can have fatal consequences. Therefore, low toxicity and stable biocompatibility are crucial for the clinical translation of NIR QDs (Ozkan Vardar et al., 2018; Aydemir et al., 2020). While NIR QDs have been extensively studied for their toxicity issues, it is important to note that preclinical studies may not always accurately predict the material’s behavior in clinical trials.

In the related research for Yan Wo’s group and Thomas Reiner’s group (Feng et al., 2019), it was discovered that PbS quantum dots, formed by Pb^2+^ and subjected to water-soluble surface modification, exhibit favorable biocompatibility. Through metabolic experiments in mice, researchers discovered that the presence of these quantum dots resulted in a decreased ion release rate and an increased clearance rate in the body. Furthermore, no deposition of PbS quantum dots was observed in the liver, spleen, and other organs. These findings indicate that these quantum dots exhibit extremely low cytotoxicity and a high rate of blood clearance, thus suggesting their potential for clinical applications. In research on fluorescent agents, it is worth considering modifying their molecular structure to reduce toxicity. This can be achieved by removing or altering specific functional groups. In addition to this, the research also emphasizes the exploration of non-toxic or low-toxic new peptide sequences and various small molecular substances during the nerve electrical signal transduction process (Hingorani et al., 2018; Gonzales et al., 2020), which are coupled with currently clinically approved fluorescent agents (such as ICG). It is crucial to carry out numerous experiments to ascertain the safety events and dosage range for its application before implementing clinical transformation.

Currently, NIR-I imaging is rapidly moving towards broad clinical application, while NIR-II imaging is entering a transitional period from basic research to more advanced applications. We believe that as fluorescent agents continue to develop and imaging systems are upgraded, the application of NIR-II imaging will be more extensive than that of NIR-I in the future. The use of NIR imaging for intraoperative nerve visualization offers several advantages. Real-time imaging and high SBR facilitate accurate nerve identification during surgery. Furthermore, NIR light has a stronger penetration ability compared to visible light. This non-invasive method does not cause additional harm to the patient and does not interfere with normal surgical procedures.

The development of optical molecular imaging techniques for surgical guidance is progressing rapidly. The fluorescent agents mentioned in the paper are summarized in Table 1. However, the identification of neural tissue using these techniques is still limited. Due to the unique properties of neural tissue, it is crucial to select a suitable fluorescent agent for visualization. An ideal neuro fluorescent agent should possess the following characteristics: ① excitation and emission wavelengths in the NIR window, ② easy perioperative administration, ③ log D between 0.5 and 3 at pH = 7.4, and molecular weight <500Da to ensure maximum BNB penetration, ④ long-term retention in neural tissue, ⑤ high SBR value; ⑥ high safety (Waterhouse, 2003; Pajouhesh and Lenz, 2005; Fagerholm, 2007; SAJI, 2017; Walsh et al., 2019). Then, we are faced with several problems. The issue at hand is that nerve-specific agents need to have a low enough molecular weight to pass through the BNB. However, for a NIR agent to achieve the excited state in the NIR region, it must have a sufficiently large conjugated system, which ultimately increases its molecular weight (Gibbs-Strauss et al., 2011). The structure-activity relationship (SAR) of neuron-specific agents has been proposed as a way to guide the synthesis of other fluorescent agent libraries based on the molecular configuration of the agent because changes in the surrounding environment can affect the imaging effect of agents (Gibbs et al., 2013).

**TABLE 1:** Florescent agents in research use.

Antigen target	References	Original agent	Class	Administration route	Probes	Fluorescent agent	Excitation wavelength (nm)	Emission wavelength (nm)	Nerve-specific
Axon	Miller et al. (2021)	FG	Carbocyanine dye	Local	-	FG	350	405	-
	Glasgow et al. (2016)	-	Neurotropic viral	Systemic	-	-	-	-	^a^
	Massaad et al. (2015)	-	Mab	Systemic	-	-	-	-	^a^
	Liu et al. (2019b)	BDA	Amine	Systemic	SPDS	BDA-CPDS	570	640	^a^
	Gonzales et al. (2020)	Hsla	Peptide	Systemic	Cy7.5	Hsla-FL	720	835	^a^
	Johnson et al. (2009)	BChE	Cholinesterase	Systemic	IRDye	BChE- IRDye	685、785	805	^a^
Pericellular membrane	Walsh et al. (2019)	Dio/Fast Dio	Carbocyanine dye	Local	-	Dio/Fast Dio	484	501	-
Myelin	Walsh et al. (2019)	FB	Carbocyanine dye	Local	-	FB	365	420	-
	Wang et al. (2010)	CMC	Coumarin derivative	Systemic	-	CMC	348、407、655	479、551、674	^a^
	Lin et al. (2014)	FIC	Coumarin derivative	Systemic	-	FIC	-	-	^a^
	Wu et al. (2006)	BDB	DSB	Systemic	-	BDB	426	506	^a^
	Liu et al. (2017)	BMB	DSB	Systemic	-	BMB	398	500	^a^
	Liu et al. (2017)	BDB-m	DSB	Systemic	-	BDB-m	385	525	^a^
	Gibbs-Strauss et al. (2011)	GE3082	DSB	Systemic	-	GE3082	414	584	^a^
	Cotero et al. (2012)	GE3111	DSB	Systemic	-	GE3111	396	594	^a^
	Cotero et al. (2015)	GE3126	DSB	Systemic	-	GE3126	395	583	^a^
Neuron	Walsh et al. (2019)	NeuroTrace	Small tolecular	Local	-	NeuroTrace	500	525	-
Neurovascular bundle	Chen et al. (2015)	ICG	Cyanine dye	Systemic	-	ICG	805	835	-
Neurilemma	Wang et al. (2020a)	Oxzine1	Oxazine derivative	Systemic	-	Oxzine1	660	680	^a^
	Wang et al. (2020a)	Oxzine4	Oxazine derivative	Systemic	-	Oxzine4	620	640	^a^
	Wang et al. (2020a)	LGW01-08	Oxazine derivative	Systemic	-	LGW01-08	640	680	^a^
	Wang et al. (2020a)	LGW03-76	Oxazine derivative	Systemic	-	LGW03-76	640	665	^a^
	Wang et al. (2020a)	LGW04-31	Oxazine derivative	Systemic	-	LGW04-31	640	670	^a^
	Wang et al. (2020a)	LGW05-75	Oxazine derivative	Systemic	-	LGW05-75	655	675	^a^
	Glasgow et al. (2016)	NP41	Peptide	Systemic	FAM	NP41-FAM	450–490	500–550	^a^
	Hingorani et al. (2018)	NP401	Peptide	Systemic	FAM	NP401-FAM	488	515	^a^
	Kleinjan et al. (2014)	PNA	Lectin	Local	Cy5	PNA-Cy5	640	680	^a^
	Kleinjan et al. (2014)	PHA-L	Lectin	Local	Cy5	PHA-L-Cy5	640	680	^a^
	Kleinjan et al. (2014)	LEL	Lectin	Local	Cy5	LEL-Cy5	640	680	^a^
	Kleinjan et al. (2014)	WGA	Lectin	Local	Cy5	WGA-Cy5	640	680	^a^

^a^ Means Yes—means No.

**FIGURE 9:**
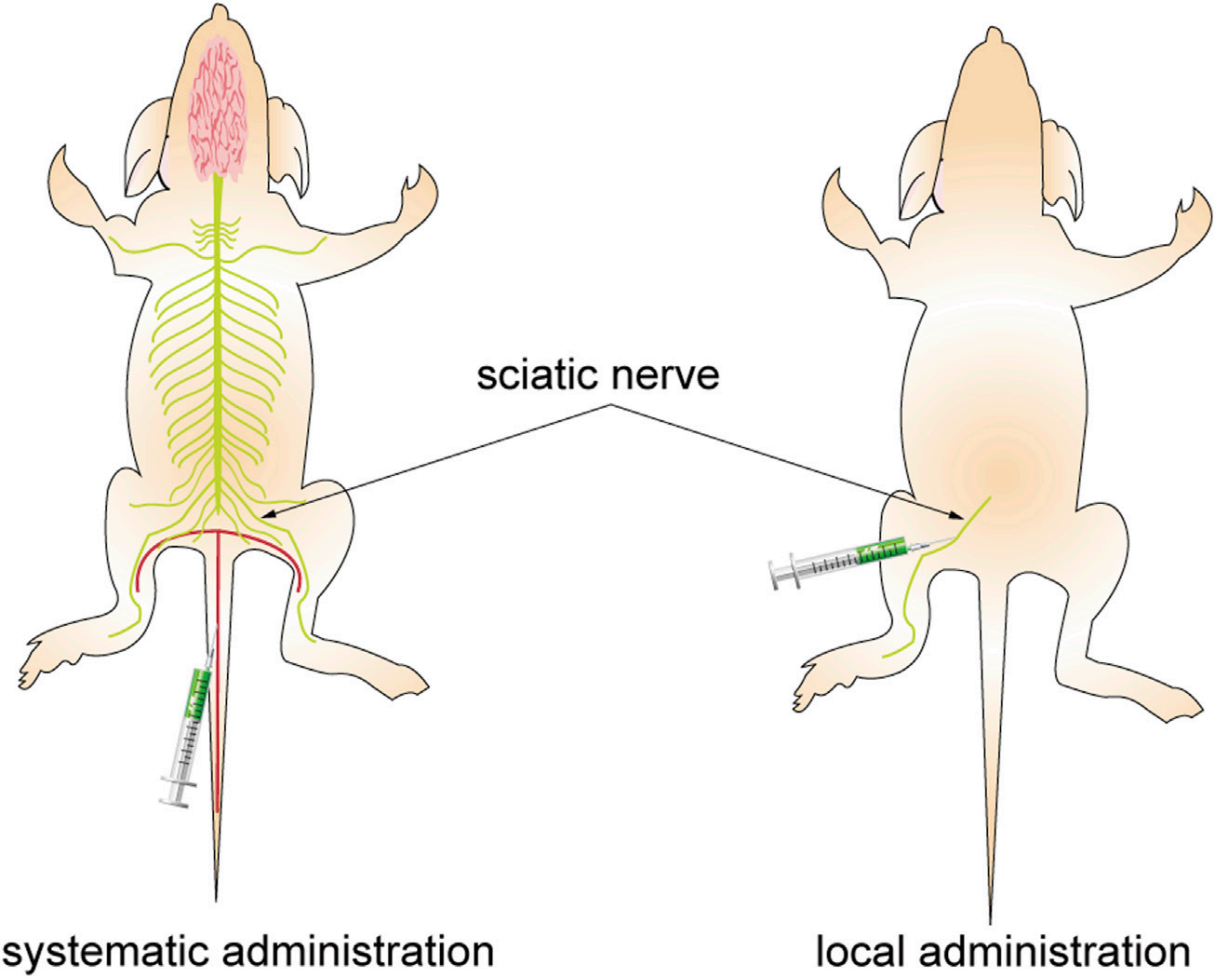
Different administration approaches. The figure on the left shows systemic administration. The agents are injected into the mice through the caudal vein, pass through the BNB, and combine with the nerve after reaching the whole body with the blood circulation. The figure on the right shows local administration. The agents are injected directly into the area to bind to the target nerve.

Various injection methods have been found to impact the resulting fluorescence, as depicted in Figure 9. While systemic administration may decrease the SBR of vital nerves, local administration at the same dosage can enhance the fluorescence signal of the neural target. Moreover, the fluorescent agent dosage and pre-injection time required for local administration to achieve the same fluorescence intensity were significantly lower than those for systemic administration. Local administration of fluorescent agents cannot effectively absorb and eliminate them through circulation, leading to potential tissue accumulation and injury due to their cytotoxicity. Therefore, to enhance nerve-specific fluorescence intensity and minimize non-specific fluorescence background signal and tissue damage, it is crucial to carefully consider the injection technique, dosage, and pre-injection period (Barth and Gibbs, 2017).

Currently, there is no standard consensus on accurately and comprehensively evaluating the effectiveness of existing fluorescent agents in clinical reports due to the use of different fluorescent agents, a small patient sample size, and varying clinical diagnoses and preoperative interventions. To fully understand the effects of neuro-specific agents, it is crucial to investigate their physicochemical characteristics such as injection dose, injection time, cytotoxicity, sensitivity, temporal and spatial resolution, penetration depth, and pharmacokinetics. Intraoperative optical data can be used to improve the process of assessing the location of the target nerve during surgery. Currently, this process is subjective and relies on the surgeon’s judgment. By analyzing the quantitative outcomes of the optical data in real-time, the location and course of the nerve can be judged based on the changes in the optical signal feedback from the instrument.

The imaging of nerves in fluorescence-guided surgery is advancing smoothly thanks to the development of fluorescent agents. This technology is expected to further improve intraoperative nerve preservation and monitoring of nerve regeneration, as well as aid in the identification of neurological illnesses. As clinical settings continue to adopt this developing technology, we can expect significant advancements in the field. In comparison to previous studies that have summarized the principles of neuro fluorescence imaging using existing fluorophores and the imaging effects of optical molecular imaging technology in central nervous system diseases, this research focuses on discussing the future development trends and advancements in new imaging equipment and fluorescent agents (Chen et al., 2020; Cen et al., 2022). The aim is to inspire and guide future researchers in this field. Moving forward, our team will continue conducting extensive research in the hopes of obtaining neuro-specific agents with highly safe and excellent imaging effects as soon as possible.
